# Perigastric Hematoma Masquerading as a Gastric Tumor

**DOI:** 10.1155/2023/9568983

**Published:** 2023-08-21

**Authors:** Kevin Yang, Nariman Hossein-Javaheri, Naren Nallapeta, Mayada Ismail

**Affiliations:** University at Buffalo, State University of New York, Buffalo, USA

## Abstract

Perigastric and intramural gastric hematomas rarely occur, with most cases associated with trauma, coagulopathy, and peptic ulcer disease. Furthermore, hematomas in the upper gastrointestinal tract are commonly located in the esophagus and duodenum. In this case report, we describe a hematoma masquerading as a gastric tumor on esophagogastroduodenoscopy (EGD) in a 54-year-old male presenting with melena. Initial computed tomography (CT) imaging suggested gastrointestinal stromal tumor (GIST) as the probable cause. We performed endoscopic ultrasound (EUS) with findings consistent with a perigastric hematoma, which aligned with the patient's diagnosis of splenic vein thrombosis (SVT) and numerous collateral vessels communicating with the hematoma. Interventional radiology (IR) was consulted for further management, although we ultimately chose a conservative approach.

## 1. Introduction

Most hematomas observed in the upper gastrointestinal tract are in the esophagus and duodenum. When they occur in or around the stomach wall, they are commonly associated with trauma, coagulopathy, and peptic ulcer disease [1]. In the absence of these risk factors, the differential diagnosis is broader and may include gastric tumor/cancer, lymphoma, or abscess. These gastric masses are often seen on EGD or CT imaging, which prompts further workup and can include EUS with biopsy, intervention by IR, and more detailed imaging. In our patient, we proceeded with EUS, which revealed a perigastric hematoma and findings of SVT with multiple collateral vessels.

## 2. Case Description

**Figure 1:**
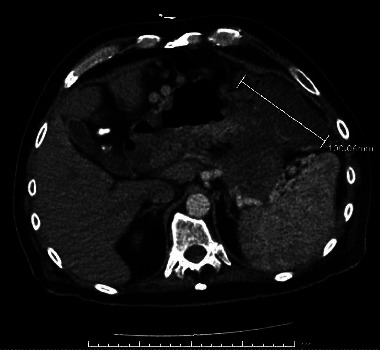
Axial CT view showing the large gastric mass.

**Figure 2:**
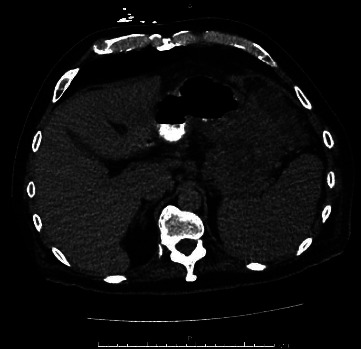
Axial CT view 2 showing the large gastric mass.

**Figure 3:**
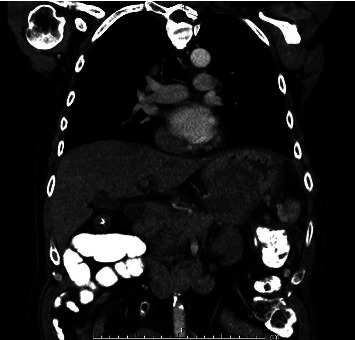
Coronal CT view of the large complex gastric mass.

**Figure 4:**
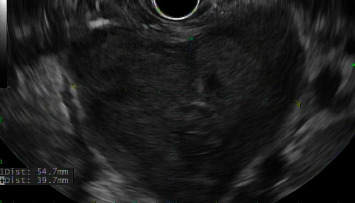
Endoscopic ultrasound showing perigastric hematoma and associated collateral vessels.

**Figure 5:**
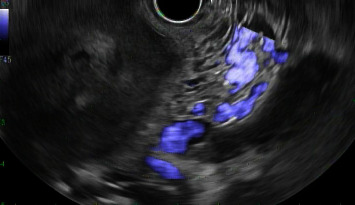
Endoscopic ultrasound view 2 showing perigastric hematoma and associated collateral vessels.

In this case report, we discuss a 54-year-old male with no relevant medical history who initially presented with abdominal pain and melena. Vital signs were stable, although hemoglobin dropped to a nadir of 5.3 g/dL. After transfusion, he underwent an EGD, which showed a large submucosal, noncircumferential mass without active bleeding in the cardia. Biopsies were taken at this time, although they were inconclusive based on the pathology review. Given the appearance of the mass, a gastric tumor/cancer was suspected. Initial CT imaging showed a large complex gastric mass extending both posterior and anterior to the gastric lumen, including a greater than 6 cm component extending posteriorly and contiguous with the distal body and tail and a greater than 10 cm component extending anteriorly contiguous with the splenic flexure colon (Figures 1–3). No cirrhotic liver changes were seen. Subsequent EUS showed type 1 isolated gastric varices in the fundus and a large intraabdominal fluid collection likely representing bleeding from collaterals, given continuity with adjacent prominent collaterals (Figures 4 and 5). Numerous collateral vessels were noted in the perigastric and perisplenic regions. The apparent etiology was SVT seen on contrast-enhanced imaging, although the patient did not have a documented history of pancreatitis or coagulopathy. IR was consulted for further management of the hematoma and SVT. Due to the location of the hematoma, we chose a conservative approach, and the patient remained stable until discharge without requiring further blood transfusions. In addition, in this case, the risks of anticoagulation outweighed the benefits.

## 3. Discussion

It can be challenging to differentiate hematomas from other gastric masses on initial imaging such as EGD and CT scans. The differential can be quite broad and includes tumor/cancer, lymphoma, and abscess. Furthermore, gastric tumors and cancer such as GISTs may also present as intramural gastric hematomas or following rupture with extensive intraabdominal hemorrhage, which can complicate the diagnosis [2, 3]. In addition, intramural gastric hematomas are uncommon since most upper gastrointestinal hematomas appear in the esophagus or the duodenum. A PubMed search for all reported adult cases of intramural gastric hematomas over the past 10 years identified only 11 reported cases. Many gastric hematomas occur via trauma, following procedures, or with coagulopathies. In our patient, the only etiology identified was SVT, which most commonly occurs in the context of pancreatitis, liver cirrhosis, abdominal malignancies, and hypercoagulable states. Moreover, SVT is a well-recognized complication of acute and chronic pancreatitis, and a few cases have attributed pancreatitis directly to the development of gastric hematomas [4–7]. It is suspected that pancreatic enzymes induce irritation of the peripancreatic vasculature including the splenic vein, resulting in coagulopathy and the formation of a hematoma. In cases of SVT, collateral vessels may form to bypass the obstructed or narrowed segment of the splenic vein. These collateral vessels develop to redirect blood flow and maintain adequate circulation to the spleen and surrounding organs. The specific mechanisms of collateral vessel formation in SVT are not fully understood, but there are a few possible pathways, which include recanalization, venous network expansion, and retrograde flow. Our patient did not have any known risk factors for developing SVT, although it is reasonable to suspect he had a prior episode of pancreatitis that was not observed. He also had a subsequent workup for malignancy, which was unremarkable. Nonetheless, the diagnosis of a perigastric hematoma should be considered in patients presenting with gastrointestinal bleeding and found to have a gastric mass, especially in patients with known varices and splenic vein thrombosis.

Treatment of SVT is far from standardized and may involve anticoagulation therapy, thrombectomy, other endovascular procedures, management of underlying conditions, such as cirrhosis and pancreatitis, and even surgery if indicated [8]. There is no standby strategy; the choice of treatment depends on individual patient characteristics and should involve a multidisciplinary approach with specialists such as gastroenterologists, interventional radiologists, or surgeons. Our case was complicated by SVT that resulted in a perigastric hematoma, which precluded utilizing anticoagulation therapy such as heparin. Given the location of the hematoma, IR decided against a more invasive approach of embolization, although this inherently comes with the risk of rebleeding [9].

To date, no definitive diagnostic approach or therapy for intramural/perigastric hematomas has been proposed. Considering the precarious nature of these hematomas, endoscopic procedures may exacerbate bleeding diatheses, particularly with biopsies or submucosal injections. Upon discovery of an intramural gastric hematoma, initial management generally requires appropriate blood and volume resuscitation and correction of any coagulopathy. It appears that most cases are managed conservatively depending on the patient's hemodynamic stability [10–12]. Conservative management in these cases essentially involves blood transfusions, symptom control, correction of underlying conditions, and serial monitoring. If no improvement is observed, subsequent management might require surgery, angiography, and vascular embolization through IR [13–15]. Specifically for pancreatitis-induced gastric hematoma, most cases were managed conservatively except for one requiring percutaneous drainage. For our patient, further evaluation from surgery and IR recommended no immediate intervention. Our patient was monitored for improvement of the hematoma and remained stable throughout discharge.

## 4. Conclusion

Perigastric/intramural gastric hematomas should be included in the differential diagnosis for patients presenting with gastrointestinal bleeding and found to have a gastric mass, particularly in those with a history of trauma, coagulopathy, and PUD. Patients with known splenic vein thrombosis and varices/collaterals warrant an even higher suspicion for hematoma. EGD and CT imaging are typically part of the initial workup; however, EUS can provide a more definitive diagnosis. In our patient, this revealed a perigastric hematoma secondary to splenic vein thrombosis with continuity to adjacent prominent collaterals. Management is typically multidisciplinary, although conservative management is often chosen, which involves serial monitoring of the hematoma, controlling underlying conditions, blood and fluid resuscitation, and symptom relief.

## Data Availability

The data used to support the findings of this study are included within the article.
